# Effects and Molecular Regulation Mechanisms of Salinity Stress on the Health and Disease Resistance of Grass Carp

**DOI:** 10.3389/fimmu.2022.917497

**Published:** 2022-06-06

**Authors:** Hong Fang, Yuan Yuan Yang, Xiao Man Wu, Si Yao Zheng, Yun Jie Song, Jie Zhang, Ming Xian Chang

**Affiliations:** ^1^ State Key Laboratory of Freshwater Ecology and Biotechnology, Institute of Hydrobiology, Chinese Academy of Sciences, Wuhan, China; ^2^ College of Advanced Agricultural Sciences, University of Chinese Academy of Sciences, Beijing, China; ^3^ Innovation Academy for Seed Design, Chinese Academy of Sciences, Wuhan, China

**Keywords:** grass carp, salinity treatments, NOD-like receptor signaling pathway, NLRs, *F. columnare* infection

## Abstract

Though some freshwater fish have been successfully cultivated in saline-alkali water, the survival rates of freshwater fish are greatly affected by different saline-alkali conditions. The mechanisms of immune adaptation or immunosuppression of freshwater fish under different saline-alkali stress remain unclear. Here, grass carp were exposed to 3‰ and 6‰ salinity for 30 days. It was observed that salinity treatments had no obvious effects on survival rates, but significantly increased the percent of unhealthy fish. Salinity treatments also increased the susceptibility of grass carp against *Flavobacterium columnare* infection. The fatality rate (16.67%) of grass carp treated with 6‰ salinity was much lower than that treated with 3‰ salinity (40%). In the absence of infection, higher numbers of immune-related DEGs and signaling pathways were enriched in 6‰ salinity-treated asymptomatic fish than in 3‰ salinity-treated asymptomatic fish. Furthermore different from salinity-treated symptomatic fish, more DEGs involved in the upstream sensors of NOD-like receptor signaling pathway, such as NLRs, were induced in the gills of 6‰ salinity-treated asymptomatic fish. However in the case of *F. columnare* infection, more immune-related signaling pathways were impaired by salinity treatments. Among them, only NOD-like receptor signaling pathway was significantly enriched at early (1 and/or 2 dpi) and late (7 dpi) time points of infection both for 3‰ salinity-treated and 6‰ salinity-treated fish. Besides the innate immune responses, the adaptive immune responses such as the production of Ig levels were impaired by salinity treatments in the grass carp infected with *F. columnare.* The present study also characterized two novel NLRs regulated by salinity stress could inhibit bacterial proliferation and improve the survival rate of infected cells. Collectively, the present study provides the insights into the possible mechanisms why the percent of unhealthy fish in the absence of infection and mortality of grass carp in the case of *F. columnare* infection were much lower in the 6‰ salinity-treated grass carp than in 3‰ salinity-treated grass carp, and also offers a number of potential markers for sensing both environmental salinity stress and pathogen.

## Highlights

1. Salinity treatments affect grass carp health.
2. Salinity treatments increase the susceptibility of grass carp against *F. columnare* infection.
3. The adverse effects of salinity stress on grass carp were much lower in the 6‰ salinity-treated fish than in 3‰ salinity-treated fish.
4. NLRs regulated by salinity treatments may act as the sensors for environmental salinity stress.
5. The inhibited IgM and NLRs may correlate with mortality of grass carp with salinity treatments and *F. columnare* infection.

## Introduction

Saline-alkali water has the characteristics of high pH, high salinity, high alkalinity, and unstable proportion of major ions, which accounts for a considerable proportion of water resources in the world. China has about 1.5 billion mu of saline-alkali land, 690 million mu of low-lying saline and alkaline water, and saltwater water lakes that account for approximately 55% of total lake area. To utilize saline-alkali water and saline-alkali land to develop aquaculture is of great significance for improving comprehensive production capacity of saline-alkali water and soil resources. Some freshwater fish, such as crucian carp (*Carassius auratus gibelio*), grass carp (*Ctenopharyngodon idell*a), bighead carp (*Hypophthalmichthys nobilis*), medaka (*Oryzias latipes*) and channel catfish (*Ictalurus punctatus*), have been successfully cultivated in saline-alkali water. In 2012, the water quality standard for aquaculture in saline-alkali land was published for the first time in China. Due to the differences of chemical components in saline-alkali water, it was divided into three levels including class I, class II and class III saline-alkali water quality. Among them, class I saline-alkali water quality was suitable for freshwater fish, shrimp and crabs (1).

The tolerance of several common freshwater fish against salinity is different. The order of salinity tolerance of 5 freshwater fish is *Carassius auratus* > *Barbus capito* > *songpu mirror carp* > *C. idell*a > *Hypophthalmichthys molitrix* (2). Different freshwater fish species have different suitable range for saline-alkali concentration. Saline-alkali stress higher or lower than this range will affect the growth and survival of fish (3). Although many studies have examined the effects of saline-alkali stress on the osmotic regulation (4–6), changes in organizational structure (5), antioxidant system (7–9), immune system (10), energy metabolism (11), growth and survival in freshwater fish species, it still remains to lack a thorough grasp for sensing mechanisms of freshwater fish under environment salinity stress and pathogens. Pattern recognition receptors (PRRs) are known to play an essential role in sensing intracellular and extracellular products of various pathogens (12). PRRs-mediated signaling pathways such as NOD-like receptor (NLR) and Toll-like receptor (TLR) signaling pathways have been found to be regulated by salinity stress (13). Therefore, the identification and characterization of PRRs and PRRs-mediated signaling pathways involved in the sensing of both saline-alkali stress and pathogens are essential for revealing the mechanisms of immune adaptation or immunosuppression of freshwater fish under different saline-alkali stress.

In the past several years, high-throughput mass spectrometry (MS)-based metabolomics and sequencing techniques have been used to identify the responses of freshwater fish species exposed to different concentrations of saline-alkali stress. For Crucian carp (*Carassius carassius*), the metabolic changes in 7 key pathways, which include phenylalanine metabolism, glycine, serine and threonine metabolism, pyruvate metabolism, tyrosine metabolism, cysteine and methionine metabolism, aminoacyl-tRNA biosynthesis and butanoate metabolism, played a role in adaptation to extremely high alkalinity (14). For Nile tilapia (*Oreochromis niloticus*) in response to alkalinity stress, comparative transcriptome analysis revealed that the most significant pathway was energy metabolism (15). For Nile tilapia in response to salinity acclimation, significant changes in amino acid metabolism and synthesis, energy material utilization, protein synthesis and degradation, oxidation, and signal transduction have been found (3). Grass carp is an economically important freshwater fish in china, and can be cultivated in class I saline-alkali water. However the molecular regulation mechanism under different saline-alkali stress is still unexplored.

*Flavobacterium columnare* is a Gram-negative bacterium causing columnaris disease in a wide-range of freshwater fish. Previous studies have shown the immune response of grass carp to *F. columnare* infection. After *F. columnare* infection, TOR signaling was significantly activated, however ERK signaling was significantly inhibited (16). In grass carp immunized with *F. columnare*, the transcription and protein levels of IgM, IgZ and pIgR were up-regulated (17). In this study, the effects of different concentrations of salinity stress on the health and disease resistance of grass carp were investigated. The transcriptomic data between the untreated control fish and salinity-treated asymptomatic or symptomatic fish, together with the transcriptomic data between the untreated control and salinity-treated fish infected with *F. columnare* for 1, 2 and 7 days, were compared and analyzed to identify PRRs and PRRs-mediated signaling pathways involved in the sensing of both saline-alkali stress and pathogens. Our data showed that salinity stress influenced multiple immune related pathways, especially for NOD-like receptor signaling pathway. Furthermore, many fish-specific NLRC3-like and NLRP-like genes were identified to be pivotal in the sensing of saline-alkali stress and *F. columnare* infection. Our study enriches the knowledge about the function of fish-specific NLRs under environment salinity stress and pathogen infection.

## Materials and Methods

### Experimental Fish

Healthy grass carps (mean weight 10 ± 1 g) were obtained from Sichuan province, China. Fish were reared in 1000 L aquarium with temperature maintained at 25 ± 2°C. Fish were acclimated for two weeks and fed with a commercial pelleted diet at 3% body weight per day through-out the study. All animal experiments were conducted in accordance with the Guiding Principles for the Care and Use of Laboratory Animals approved by the Institute of Hydrobiology, Chinese Academy of Sciences.

### Cells, Bacterial Strain and Antibodies

CIK (*C. idellus* kidney) cells were grown in minimum essential medium (MEM) supplemented with 10% FBS. The wild type *F. columnare* strain and monoclonal antibodies against grass carp IgM, IgD and IgZ were obtained from professor Pin Nie’s lab (Institute of hydrobiology, Chinese academy of sciences). The anti-GAPDH monoclonal antibody (#60004-1-Ig) was purchased from Proteintech. Goat-anti-mouse Ig-HRP conjugate secondary antibody (#31430) was purchased from invitrogen.

### Salinity Treatment and Infection of Grass Carp

The salt concentrations (salinity) were prepared from a commercially available sea salt (DPZ301). According to the salinity of natural tidal flats (2-7‰), 330 fish were randomly divided into three groups, with the salinity concentration set as 0‰ (the untreated control group), 3‰ and 6‰ (pH 7.2-7.4). The healthy condition and the numbers of surviving fish were monitored daily for 30 days. During this period, new aquaculture water and sea salt were replaced regularly, once a week or so. Survival curves and percent of healthy fish were generated by GraphPad Prism 7. The log-rank test was used to test differences in survival rate and percent of healthy fish between the untreated control and salinity-treated groups. After 30 days of salinity stress, the gill tissues from three individuals each group were collected, and stored at -80°C used for transcriptome sequencing. For salinity-treated groups of 3‰ and 6‰, the gill tissues were separately collected both from asymptomatic and symptomatic fish.

After 30 days of salinity stress, 35 fish per group in triplicate were infected with 2.5×10^5^ pfu/ml *F. columnare* for 4 h in total volume of 35 L aquarium, and next maintained in 70 L barrel. At 1, 2 and 7 d post injection (dpi), 3 fish from each group were anaesthetized in 0.05% 2-phenoxyethanol, and the gills were collected for transcriptome sequencing. The gills collected at 2 dpi were also used for measuring bacterial burden, Western Blotting and quantitative real-time PCR (qRT-PCR) verification. For survival assay, the numbers of surviving fish were counted daily for 7 days. Survival curves were generated by GraphPad Prism 7. The log-rank test was used to test differences in survival between the control and salinity-treated groups infected with *F. columnare*.

### Bacterial Load

The gills from the control and salinity-treated groups infected with *F. columnare* were rinsed and lysed in 500 μL of PBS. Serial dilutions of homogenates were plated onto Shieh agar, and colony counts were performed after 24 h incubation at 28°C. The bacterial concentration was normalized with respect to the weight (in mg) of each sample, and calculated in CFU/ml.

### Histopathological Examination

After 30 days of salinity stress, the gill tissues of grass carp in the untreated control group and gill tissues from symptomatic and asymptomatic grass carp in 3‰ and 6‰ salinity-treated groups were taken and fixed in 4% paraformaldehyde solution at 4°C for 24h. The fixed gill tissues were processed through the conventional paraffin embedding technique, and sliced into 5 μm-thick tissue sections. Then the slides from 3 fish per group were subjected to Hematoxylin and eosin staining, sealed with neutral gum, observed and photographed with a Leica DM4 B upright microscope.

### RNA Isolation, cDNA Library Construction and Illumina Deep Sequencing

Total RNA was isolated using the TRIzol^®^ Reagent (Invitrogen) from gill tissues including the untreated control fish (con), 3‰-treated asymptomatic fish, 3‰-treated symptomatic fish, 6‰-treated asymptomatic fish, 6‰-treated symptomatic fish, the untreated control fish infected with *F. columnare* for 1 day (named as con at 1 dpi or G1dcon), 3‰-treated fish infected with *F. columnare* for 1 day (named as 3‰-treatment at 1 dpi or G1dP3), 6‰-treated fish infected with *F. columnare* for 1 day (named as 6‰-treatment at 1 dpi or G1dP6), the untreated control fish infected with *F. columnare* for 2 day (named as con at 2 dpi or G2dcon), 3‰-treated fish infected with *F. columnare* for 2 day (named as 3‰-treatment at 2 dpi or G2dP3), 6‰-treated fish infected with *F. columnare* for 2 day (named as 6‰-treatment at 2 dpi or G2dP6), the untreated control fish infected with *F. columnare* for 7 day (named as con at 7 dpi or G7dcon), 3‰-treated fish infected with *F. columnare* for 7 day (named as 3‰-treatment at 7 dpi or G7dP3), 6‰-treated fish infected with *F. columnare* for 7 day (named as 6‰-treatment at 7 dpi or G7dP6). CDNA library construction and illumina deep sequencing were performed according to the methods from our previous report (18). The TruseqTM RNA sample prep Kit (Illumina, California, USA) was used for creating mRNA-seq libraries. The clean high quality paired-end reads from all 15 samples for the uninfected grass carp and 27 samples for the grass carp infected with *F. columnare* were processed to produce a *de-novo* assembly with Trinity, respectively. To identify differentially expressed genes between the control and salinity-treated groups without or with the infection of *F. columnare*, the expression levels were measured by using numbers of fragments per kilobase of transcript per million fragments sequenced (FPKM). The raw reads were deposited in the SRA-NCBI database with the following accession numbers: GSE185170 for 15 samples from the untreated control fish, 3‰-treated asymptomatic fish, 3‰-treated symptomatic fish, 6 ‰-treated asymptomatic fish and 6‰-treated symptomatic fish; GSE185641 for 27 samples from con at 1 dpi (G1dcon), 3‰-treatment at 1 dpi (G1dP3), 6‰-treatment at 1 dpi (G1dP6), con at 2 dpi (G2dcon), 3‰-treatment at 2 dpi (G2dP3), 6‰-treatment at 2 dpi (G2dP6), con at 7 dpi (G7dcon), 3‰-treatment at 7 dpi (G7dP3) and 6‰-treatment at 7 dpi (G7dP6).

### qRT-PCR Validation of the RNA-Seq Analysis

The same RNA samples at 2 dpi from the gill transcriptome of the control and salinity-treated groups infected with *F. columnare* were used for qRT-PCR validation of the RNA-seq analysis. The concentration of total RNA was determined by using the spectrophotometer (NanoDrop 2000; Thermo). RNase-free DNase I (Thermo) was used to remove genomic DNA remnants at 37°C for 30 min. The cDNA was synthesized using the RevertAid™ First Strand cDNA Synthesis Kit (Thermo Fisher Scientific) according to the manufacturer’s instructions. qRT-PCR analysis was performed to validate the DEGs involved in NACHT containing proteins on ABI Prism 7000 under the following conditions: 3 min at 95°C, followed by 45 cycles of 15 s at 94°C, 15 s at 56-60°C, and 30 s at 72°C. All reactions were performed in triplicate in a 96 well plate and the mean value recorded. Those DEGs for validation include NLRP12 (CI01000353_00909159_00930428.path1), NLRC3 (CI01000433_00039954_00060508.path1), NLRC3 (CI01000140_02095891_02110510.path1), NLRC3 (CI01117969_00000011_00000940.path1), NLRC3 (CI01000092_03702024_03712418.path1), NLRP3-like (CI01000152_01231478_01232527.path1), NLRC3 (CI01000397_00142266_00144610.path1), NLRC3 (Ctenopharyngodon_idella_newGene_4697), NLRP12 (CI01000140_02047275_02055290.path1), NLRP3-like (CI01000070_04844701_04867804), NLRC3 (CI01000358_00550693_00557903.path1) and NLRC3 (CI01000427_00019738_00058058.path1). The housekeeping gene β-actin and EF-1α were used for normalizing cDNA amounts. The primers specific for the interested DEGs were listed in **Table 1**.

**Table 1:** Primer information.

Name	Sequence	Application
CI01000353_00909159_00930428.path1 NLRP12F	GGACCTTTTCCTTCGTTTCC	qRT-PCR
CI01000353_00909159_00930428.path1 NLRP12R	AGGAGAGGGATTTTCCCTGA	
CI01000433_00039954_00060508.path1 NLRC3F	GCCCGTGATTAAAGAATCCA	
CI01000433_00039954_00060508.path1 NLRC3R	GCCACAATCCCTCAACCTC	
CI01000140_02095891_02110510.path1 NLRC3F	GCCAGGACTGGAACTCAAAA	
CI01000140_02095891_02110510.path1 NLRC3R	ACCAGACCAGACCACTGAGC	
CI01117969_00000011_00000940.path1 NLRC3F	ACATCTCAGCGTCCAGGAGT	
CI01117969_00000011_00000940.path1 NLRC3R	CAGAGGAAAAGGTCCAGGTG	
CI01000092_03702024_03712418.path1 NLRC3F	TGATGTCTTTTCGGCATCAG	
CI01000092_03702024_03712418.path1 NLRC3R	ACCCTCATGCTGTTCTCCAC	
CI01000152_01231478_01232527.path1 NLRP3-like F	CACGACGAATCCTTCTCACA	
CI01000152_01231478_01232527.path1 NLRP3-like R	CCTGTCGTGGTTCTACAGCA	
CI01000397_00142266_00144610.path1 NLRC3F	GTCAGTGAAGGGTCGGTGTT	
CI01000397_00142266_00144610.path1 NLRC3R	CTTCCGGACAGTTTCCATGT	
Ctenopharyngodon_idella_newGene_4697 NLRC3F	GAAGTGCTGCCCTCTATTGC	
Ctenopharyngodon_idella_newGene_4697 NLRC3R	GGGAACCAGGTGTCTCTTGA	
CI01000140_02047275_02055290.path1 NLRP12F	CCAGCCTCATGAACCAGATT	
CI01000140_02047275_02055290.path1 NLRP12R	TGCAGCTTCACAGCTTCACT	
CI01000070_04844701_04867804 NLRP3-likeF	GGAAAGTGGCTTTTCAGCAG	
CI01000070_04844701_04867804 NLRP3-likeR	AAGCCGCTAGATGTTCCTGA	
CI01000358_00550693_00557903.path1 NLRC3F	GCAGCAACAGATGAAAGCAA	
CI01000358_00550693_00557903.path1 NLRC3R	AACACCGACCCTTCACTGAC	
CI01000427_00019738_00058058.path1NLRC3F	GCCCGTGATTAAAGAATCCA	
CI01000427_00019738_00058058.path1 NLRC3R	AAGCCGCTAGATGTTCCTGA	
EF1αF	CAGCACAAACATGGGCTGGTTC	
EF1αR	ACGGGTACAGTTCCAATACCTCCA	
β-actinF	CACTGTGCCCATCTACGAG	
β-actinR	CCATCTCCTGCTCGAAGTC	
NLRP12-like 1F	CGGAATTCATGGAGATGCGGATTTTG	Ligated to p3xFLAG-CMV™-14 vector
NLRP12-like 1R	CGGGATCCTGCATTTGGCAGATCCTG	
NLRP12-like 2F	GACAGATCTGATGCCTGCGATTCAGCCACAG	
NLRP12-like 2R	CGGGGTACCGACATATGTATCCCCAGTTTATAC	

### Western Blotting Validation of the RNA-Seq Analysis

The gills samples collected at 2 dpi from the control and salinity-treated groups infected with *F. columnare* were used for Western Blotting validation of the RNA-seq analysis. After washed with ice-cold PBS buffer, the gills were lysed in RIPA buffer containing Halt Protease Inhibitor Cocktail (Thermo Scientific, Prod# 1860932). After incubation on ice for 30 min, lysates were collected and centrifuged at 10,000 × g at 4°C for 15 min. Total lysates were subjected to 10% SDS-PAGE and transferred to PVDF membranes, followed by blocking with 5% nonfat milk in Tris-buffered saline-Tween (TBST) for 1 h. The membrane was washed, and then incubated with primary antibody overnight at 4°C. The primary antibodies including anti-GAPDH (1: 5000), anti-IgD (1: 2000), anti-IgM (1: 5000) or anti-IgZ (1: 2000) were used. After washing with TBST, the membrane was next incubated with Goat-anti-mouse Ig-HRP conjugate secondary antibody (1: 5000) for 1 h at room temperature. The bands were detected using Pierce ECL Western Blotting Substrate and ECL Western blot system (LAS-4000mini).

### Antibacterial Activity for NACHT Containing Proteins

Based on transcriptome sequencing, the forward and reverse primers NLRP12-like 1F/NLRP12-like 1R and NLRP12-like 2F/NLRP12-like 2R were used for cloning the open reading frame (ORF) of Ctenopharyngodon_idella_newGene_9152 (named as NLRP12-like 1) and CI01000353_00909159_00930428.path1 (named as NLRP12-like 2), and then inserted into p3×FLAG-CMV™-14 Expression Vector (Invitrogen).

For *in vitro* antibacterial analysis, CIK cells seeded overnight in 24-well plates at 2×10^5^ cells per well were transfected with 300 ng p3×FLAG, NLRP12-like 1 or NLRP12-like 2 using Lipofectamine™ 2000 (Invitrogen). After 24 h post-transfection, CIK cells were infected with *F. columnare* at a multiplicity of infection (MOI) of 50 in serum-free M199 medium for 1.5 h. Then, the supernatants and cells were collected at 6, 24 and 48 hpi. The mixture was diluted with Shieh medium and plated onto Shieh agar to calculate bacterial CFU (colony-forming units) by standard plate count method.

### Cell Viability Assay Mediated by NACHT Containing Proteins

Cell viability was determined *via* CCK-8 assay (Beyotime, Wuhan, China). CIK cells seeded overnight in 24-well plates at 2×10^5^ cells per well were transfected with 300 ng p3×FLAG, NLRP12-like 1 or NLRP12-like 2 using Lipofectamine™ 2000. After 24 h post-transfection, CIK cells were infected with *F. columnare* at a MOI of 50 for 1.5 h. At 6, 24 and 48 hpi, the cells were collected, washed with PBS, and then suspended in a working solution containing 10 µL CCK-8 reagent and 90 µL serum-free M199 medium for 3 h. The absorbance at 450 nm was measured by a PerkinElmer’s EnSpire Multilabel Plate Reader.

### Statistical Analysis

Statistical analysis and graphs were performed and produced using Graphpad Prism 7.0 software. Data from qRT-PCR are presented as mean and SEM. The significance of data was analyzed by Student’s t-test (**p < 0.01).

## Results

### Effects of Salinity Stress on Health of Grass Carp

**Figure 1:**
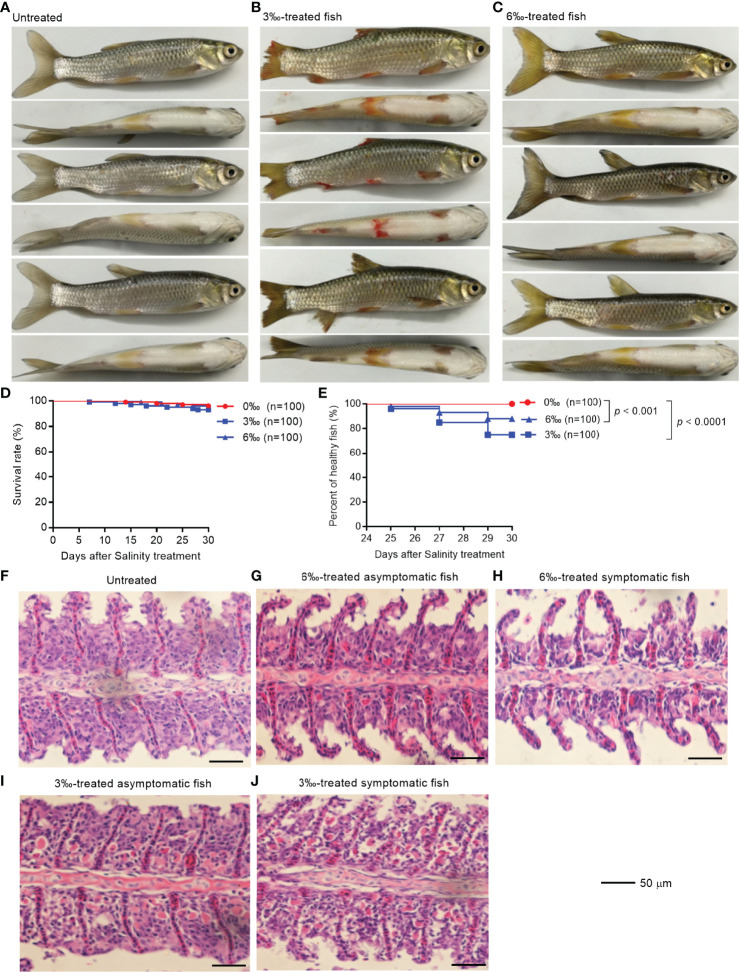
Salinity treatments impair the health of grass carp. **(A)** The untreated grass carp. **(B)** The pathological symptoms of 3‰ salinity-treated grass carp. **(C)** The pathological symptoms of 6‰ salinity-treated grass carp. **(D)** Effects of salinity treatments on the survival rate of grass carp. **(E)** Effects of salinity treatments on the percent of healthy grass carp. **(F)** Histopathological staining of gill tissue from the untreated grass carp. **(G)** Histopathological staining of gill tissue from the 6‰ salinity-treated asymptomatic fish. **(H)** Histopathological staining of gill tissue from the 6‰ salinity-treated symptomatic fish. **(I)** Histopathological staining of gill tissue from the 3‰ salinity-treated asymptomatic fish. **(J)** Histopathological staining of gill tissue from the 3‰ salinity-treated symptomatic fish.

To explore health conditions of farmed fish under saline-alkali stress conditions, grass carp were selected for salinity tolerance analysis. The observations revealed that the grass carp from salinity-treated groups were damaged to varying degrees after 25 days treatment, especially for 3‰ salinity-treated group (**Figures 1A–C**). The fish body damage in 3‰ salinity-treated group was the most serious, with fish scales falling off, fin ulceration and bleeding (**Figure 1B**). The fish in 6‰ salinity-treated group showed slight symptoms with fish scales falling off and fin ulceration (**Figure 1C**). Compared with the untreated control fish, there was no significant change in survival rates for 3‰ or 6‰ salinity-treated group (**Figure 1D**), however the percent of unhealthy fish significantly increased for 3‰ or 6‰ salinity-treated group (**Figure 1E**).

The histopathological changes in the gills of grass carp with different salinity treatments were observed by hematoxylin-eosin staining. The gills from the untreated control and 6‰ salinity-treated asymptomatic fish showed normal structures of lamellae (**Figures 1F, G** **)**. In the gills from 6‰ salinity-treated symptomatic fish, the cell mass between lamellae (ILCM) was decreased relative to control fish (**Figure 1H**). Under the 3‰ salinity stress, the gills showed obvious lamellar synechiae and the higher numbers of plasma cells, especially for 3‰ salinity-treated symptomatic fish (**Figures 1I, J** **)**.

### Analysis of Differentially Expressed Genes (DEGs)

**Figure 2:**
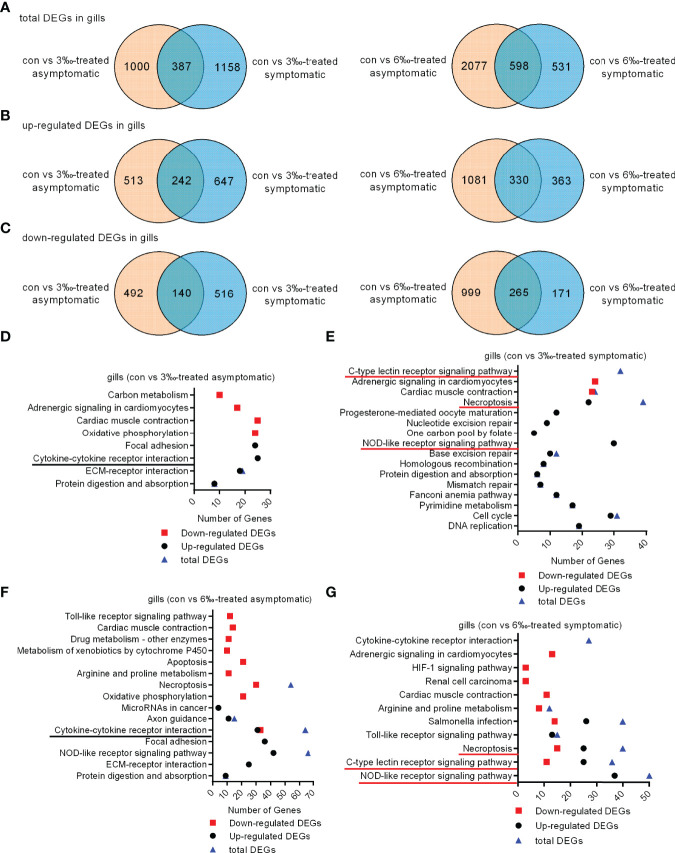
Differentially expressed genes and significantly enriched KEGG pathways in grass carp treated with 3‰ or 6‰ salinity. **(A)** Venn diagrams showing overlaps of total DEGs in gill samples from salinity-treated asymptomatic and symptomatic fish. **(B)** Venn diagrams showing overlaps of up-regulated DEGs in gill samples from salinity-treated asymptomatic and symptomatic fish. **(C)** Venn diagrams showing overlaps of down-regulated DEGs in gill samples from salinity-treated asymptomatic and symptomatic fish. **(D)** The significantly enriched KEGG pathways in the 3‰-treated asymptomatic fish. **(E)** The significantly enriched KEGG pathways in the 3‰-treated symptomatic fish. **(F)** The significantly enriched KEGG pathways in the 6‰-treated asymptomatic fish. **(G)** The significantly enriched KEGG pathways in the 6‰-treated symptomatic fish. The common immune-related signaling pathways for 3‰ and 6‰-treated asymptomatic fish are underlined in black, and in red for 3‰ and 6‰-treated symptomatic fish.

To elucidate the gene expression pattern under different salinity treatments, we firstly compared the numbers of DEGs among the untreated control, 3‰ and 6‰ salinity-treated groups. For transcriptomic analysis of 15 samples, total 96.09 Gb clean data were obtained. The clean data for each sample was more than 5.87 Gb, and the percentage of Q30 value was above 91.87%. A total of 1387 (755 up- and 632 down-regulated), 1545 (889 up- and 656 down-regulated), 2675 (1411 up- and 1264 down-regulated), 1129 (693 up- and 436 down-regulated), 631 (365 up- and 266 down-regulated), 1551 (795 up- and 756 down-regulated) and 355 (231 up- and 124 down-regulated) genes were differently expressed with Fold Change ≥ 1.5 and *p* < 0.05 in con vs 3‰-treated asymptomatic fish, con vs 3‰-treated symptomatic fish, con vs 6‰-treated asymptomatic fish, con vs 6‰-treated symptomatic fish, 3 ‰-treated fish vs 6‰-treated fish, 3‰-treated asymptomatic fish vs 6‰-treated asymptomatic fish, and 3‰-treated symptomatic fish vs 6‰-treated symptomatic fish, respectively (**Table 2**). For total DEGs, 387 and 598 common DEGs were present in 2 comparisons for 3‰ and 6‰ salinity-treated fish, respectively (**Figure 2A**). For up-regulated DEGs, 242 and 330 common DEGs were present in 2 comparisons for 3‰ and 6‰ salinity-treated fish, respectively (**Figure 2B**). For down-regulated DEGs, 140 and 265 common DEGs were present in 2 comparisons for 3‰ and 6‰ salinity-treated fish, respectively (**Figure 2C**).

**Table 2:** The number of DEGs among the untreated control, 3‰ and 6‰ salinity-treated groups.

DEG Set	DEG Number	up-regulatedNumber	down-regulatedNumber
con vs 3‰-treated asymptomatic fish	1,387	755	632
con vs 3‰-treated symptomatic fish	1,545	889	656
con vs 6‰-treated asymptomatic fish	2,675	1,411	1,264
con vs 6‰-treated symptomatic fish	1,129	693	436
3‰-treated fish vs 6‰-treated fish	631	365	266
3‰-treated asymptomatic fish vs 6‰-treated asymptomatic fish	1,551	795	756
3‰-treated symptomatic fish vs 6‰-treated symptomatic fish	355	231	124

KEGG pathway enrichment analyses were conducted for 4 comparisons. For con vs 3‰-treated asymptomatic fish, 8 significantly enriched KEGG pathways were revealed, which included 1 pathway (cytokine-cytokine receptor interaction) involved in immune system for up-regulated DEGs (**Figure 2D**). For con vs 3‰-treated symptomatic fish, 13 pathways were significantly enriched for up-regulated DEGs. Among them, 2 pathways including NOD-like receptor signaling pathway (30 DEGs) and necroptosis (22 DEGs) were involved in immune response. C-type lectin receptor signaling pathway was significantly enriched only for total DEGs (**Figure 2E**). For con vs 6‰-treated asymptomatic fish, 5 pathways among 15 significantly enriched KEGG pathways were involved in immune response, which included cytokine-cytokine receptor interaction (31 up-regulated and 33 down-regulated DEGs), necroptosis (24 up-regulated and 30 down-regulated DEGs), apoptosis (21 down-regulated DEGs), toll-like receptor signaling pathway (12 down-regulated DEGs) and NOD-like receptor signaling pathway (42 up-regulated and 24 down-regulated DEGs) (**Figure 2F**). For con vs 6 ‰-treated symptomatic fish, 6 pathways among 11 significantly enriched KEGG pathways were involved in immune response, which included NOD-like receptor signaling pathway (37 up-regulated and 13 down-regulated DEGs), c-type lectin receptor signaling pathway (25 up-regulated and 11 down-regulated DEGs), necroptosis (25 up-regulated and 15 down-regulated DEGs), toll-like receptor signaling pathway (13 up-regulated and 2 down-regulated DEGs), and salmonella infection (26 up-regulated and 14 down-regulated DEGs) (**Figure 2G**). Cytokine-cytokine receptor interaction was significantly enriched only for total DEGs. Collectively, these data suggest that the cytokine-cytokine receptor interaction pathway is significantly enriched both for 3 ‰-treated and 6 ‰-treated asymptomatic fish, c-type lectin receptor signaling, necroptosis and NOD-like receptor signaling pathways enriched both for 3 ‰-treated and 6 ‰-treated symptomatic fish.

### Salinity Treatments Increase Susceptibility to *F. columnare* Infection

**Figure 3:**
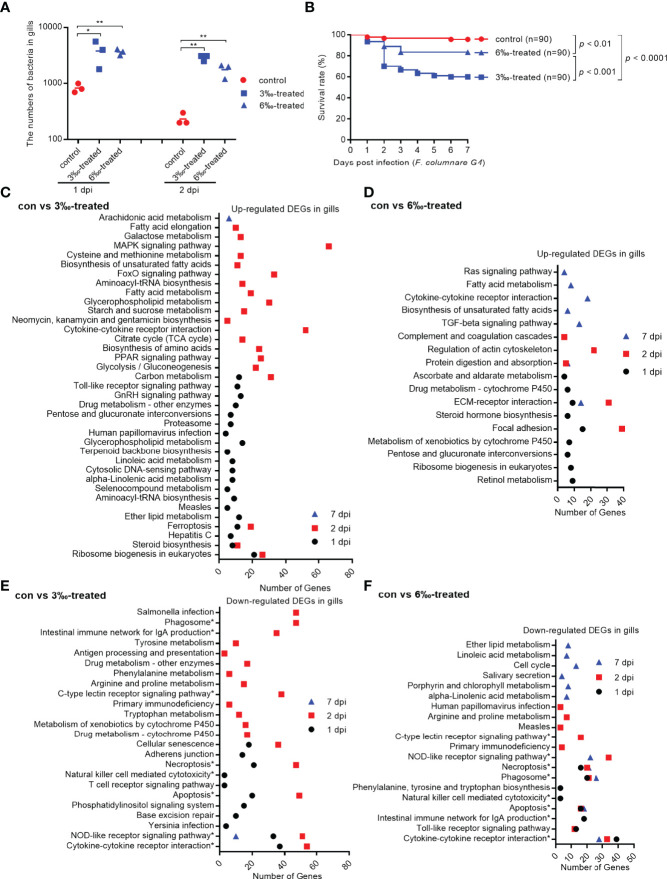
Salinity treatments increase susceptibility to *F*. *columnare* infection. **(A)** The effect of salinity treatments on the bacteria proliferation. **(B)** The effect of salinity treatments on the larvae survival in response to *F*. *columnarae* infection. **(C)** The significantly enriched KEGG pathways for up-regulated DEGs in gills collected at 1, 2 and 7 dpi from the 3‰-treated grass carp infected with *F*. *columnarae*. **(D)** The significantly enriched KEGG pathways for up-regulated DEGs in gills collected at 1, 2 and 7 dpi from the 6‰-treated grass carp infected with *F*. *columnarae*. **(E)** The significantly enriched KEGG pathways for down-regulated DEGs in gills collected at 1, 2 and 7 dpi from the 3‰-treated grass carp infected with *F*. *columnarae*. **(F)** The significantly enriched KEGG pathways for down-regulated DEGs in gills collected at 1, 2 and 7 dpi from the 6‰-treated grass carp infected with *F*. *columnarae*.

To examine the effect of salinity stress on the disease resistance of grass carp against bacterial infection, the grass carp treated by 3‰ or 6‰ salinity for 30 days were infected with *F. columnarae.* The results from bacterial colony counting showed that salinity treatments significantly increased the proliferation of *F. columnarae in vivo* at 1 and 2 dpi. The bacterial loads in 3‰ salinity-treated group was the highest (**Figure 3A**). In consistent with bacterial colony counting, salinity treatments significantly impaired the survival of grass carp infected with *F. columnarae.* Survival was more than 95% in the untreated control group, however decreased to 83.3% for 6‰ salinity-treated group, and 60% for 3‰ salinity-treated group (**Figure 3B**).

In order to reveal the possible mechanism that salinity treatments increase susceptibility to *F. columnare* infection, the gills from the untreated control and salinity-treated groups infected with *F. columnare* collected at 1, 2 and 7 dpi were used for transcriptome sequencing. For transcriptomic analysis of 27 samples, total 177.79 Gb clean data were obtained. The clean data for each sample was more than 5.73 Gb, and the percentage of Q30 value was above 93.70%. The DEGs (4152) were the highest for con at 2 dpi vs 3‰-treatment at 2 dpi, with 2103 up-regulated and 2049 down-regulated DEGs. The DEGs (2034) were the second highest for 3‰-treatment at 2 dpi vs 6‰-treatment at 2 dpi, with 1271 up-regulated and 763 down-regulated DEGs. The numbers of DEGs (356) were the lowest for con at 7 dpi vs 3‰-treatment at 7 dpi, with 195 up-regulated and 161 down-regulated DEGs (**Table 3**).

**Table 3:** The number of DEGs among the untreated control and salinity-treated groups infected with *F. columnare* collected at 1, 2 and 7 dpi.

DEG Set	DEG Number	up-regulated Number	down-regulated Number
con at 1 dpi vs 3‰-treatment at 1 dpi	1,765	793	972
con at 1 dpi vs 6 ‰-treatment at 1 dpi	1,110	419	691
3‰-treatment at 1 dpi vs 6‰-treatment at 1 dpi	421	207	214
con at 2 dpi vs 3‰-treatment at 2 dpi	4,152	2,103	2,049
con at 2 dpi vs 6‰-treatment at 2 dpi	1,379	666	713
3‰-treatment at 2 dpi vs 6‰-treatment at 2 dpi	2,034	1,271	763
con at 7 dpi vs 3‰-treatment at 7 dpi	356	195	161
con at 7 dpi vs 6‰-treatment at 7 dpi	1,416	672	744
3‰-treatment at 7 dpi vs 6‰-treatment at 7 dpi	1,306	583	723

We focused on immune-related signaling pathways. For up-regulated DEGs in gills, cytokine-cytokine receptor interaction pathway was significantly enriched for con vs 3‰-treated group only at 2 dpi, whereas at 7 dpi for con vs 6‰-treated group. Furthermore, MAPK signaling, Toll-like receptor signaling and cytosolic DNA-sensing pathways were also enriched for con vs 3‰-treated group at 1 or 2 dpi, but not for con vs 6‰-treated group (**Figures 3C, D** **)**.

More immune-related signaling pathways were impaired by salinity treatments. Eight signaling pathways including phagosome, intestinal immune network for IgA production, C-type lectin receptor signaling, necroptosis, natural killer cell mediated cytotoxicity, apoptosis, NOD-like receptor signaling and cytokine-cytokine receptor interaction were significantly enriched both for con vs 3‰-treated and con vs 6‰-treated groups. Among these 8 immune-related signaling pathways, only NOD-like receptor signaling pathway was significantly enriched at early (1 and/or 2 dpi) and late (7 dpi) times of infection both for con vs 3‰-treated and con vs 6‰-treated groups. In addition, the numbers of significantly enriched immune-related pathways varied greatly between the early and late times of infection for con vs 3‰-treated group, but remained a stable change between the early and late times of infection for con vs 6‰-treated group. At 1 dpi, 6 and 7 immune-related signaling pathways were enriched for con vs 3‰-treated and con vs 6‰-treated groups, respectively. At 2 dpi, 9 and 7 immune-related signaling pathways were enriched for con vs 3‰-treated and con vs 6‰-treated groups, respectively. At 7 dpi, 1 and 5 immune-related signaling pathways were enriched for con vs 3‰-treated and con vs 6‰-treated groups, respectively (**Figures 3E, F** **)**.

### Salinity Treatments Are Involved in the Regulation of Multiple NLRs in the Absence of Infection or Bacterial Infection

**Figure 4:**
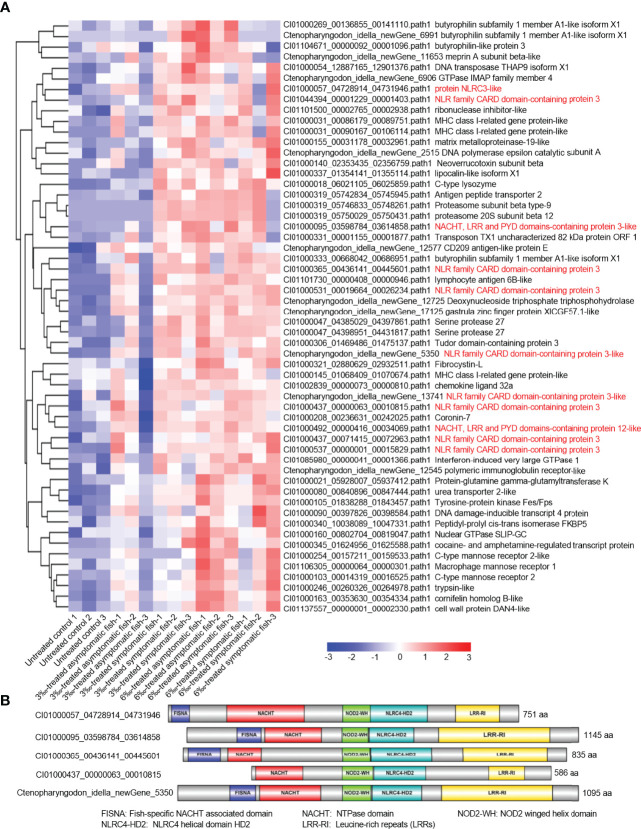
The DEGs involved in NOD-like receptor signaling pathway and regulated by salinity stress in the absence of infection. **(A)** The gene cluster for DEGs involved in the NOD-like receptor signaling pathway in gills from con vs 3‰-treated symptomatic fish, con vs 6‰-treated asymptomatic fish and con vs 6‰-treated symptomatic fish. A color key denotes the gradient scale of gene expression from low (blue) to high (red) degrees. Those NLR genes are highlighted in red. **(B)** Domain diagrams for 5 differentially expressed NLRs with the complete open reading frame.

Transcriptome analysis showed that NOD-like receptor signaling pathway was significantly enriched for salinity-treated groups in the absence of infection or *F. columnarae* infection. To clear which genes of NOD-like receptor signaling pathway were regulated by salinity, all DEGs involved in NOD-like receptor signaling pathway from con vs 3‰-treated symptomatic fish, con vs 6 ‰-treated asymptomatic fish and con vs 6‰-treated symptomatic fish (**Figures 2E–G**; **Supplementary Figures 1, 2**) were firstly picked out for analysis. After getting rid of some same sequences, a total of 56 distinct genes involved in NOD-like receptor signaling pathway were up-regulated by salinity. Among them, 11 DEGs (19.6%) are NLRC3-like genes (**Figure 4A**). Sequence analysis revealed that these 5 nucleotide sequences including CI01000057_04728914_04731946, CI01000095_03598784_03614858, CI01000365_00436141_00445601, CI01000437_00000063_00010815 and Ctenopharyngodon_idella_newGene_5350 encode the complete open reading frame. Interesting, 4 NLRC3-like genes among the 5 NLRC3-like genes contain FISNA (fish-specific NACHT associated) domain and lack CARD (caspase recruitment domain) (**Figure 4B**).

**Figure 5:**
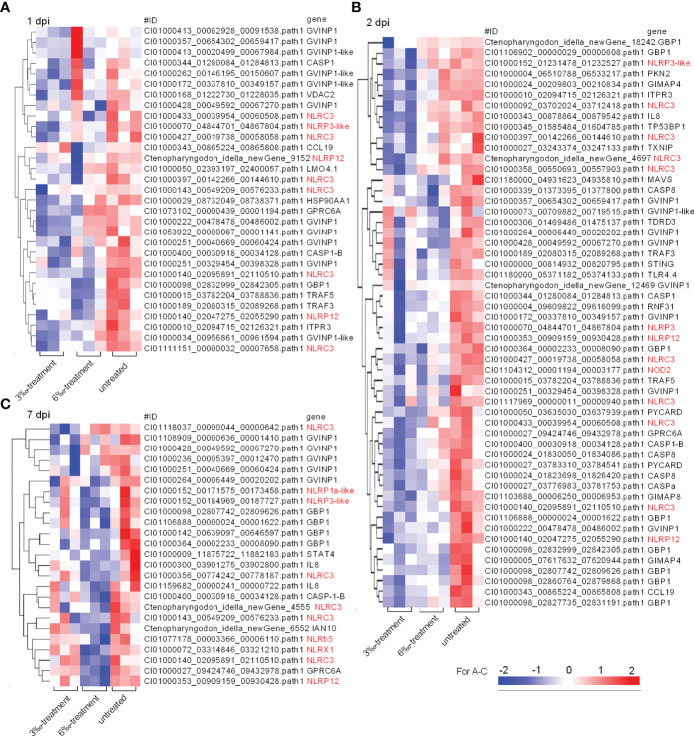
The DEGs involved in NOD-like receptor signaling pathway and regulated by salinity stress in the case of *F*. *columnare* infection. **(A)** The gene cluster for DEGs involved in the NOD-like receptor signaling pathway in gills collected at 1 dpi. **(B)** The gene cluster for DEGs involved in the NOD-like receptor signaling pathway in gills collected at 2 dpi. **(C)** The gene cluster for DEGs involved in the NOD-like receptor signaling pathway in gills collected at 7 dpi. Those NLR genes are highlighted in red. A color key denotes the gradient scale of gene expression from low (blue) to high (red) degrees.

Then, these DEGs involved in NOD-like receptor signaling pathway from the untreated control and salinity-treated groups infected with *F. columnare* were picked out for analysis. At 1 dpi, the transcription of 31 DEGs were impaired by salinity, and 9 DEGs (29%) were NLRs (**Figure 5A**). At 2 dpi, the transcription of 54 DEGs were impaired by salinity, and 13 DEGs (24.1%) were NLRs (**Figure 5B**). At 7 dpi, the transcription of 25 DEGs were impaired by salinity, and 10 DEGs (40%) were NLRs (**Figure 5C**). Furthermore, salinity treatments also significantly impaired the transcription of some genes connected to interferon responses, such as GVINP1, GVINP1-like, GBP1, TRAF3, TRAF5, MA*VS* and so on (**Figure 5**).

### Salinity Treatments Impaired the Innate and Adaptive Immune Responses in the Case of Bacterial Infection

**Figure 6:**
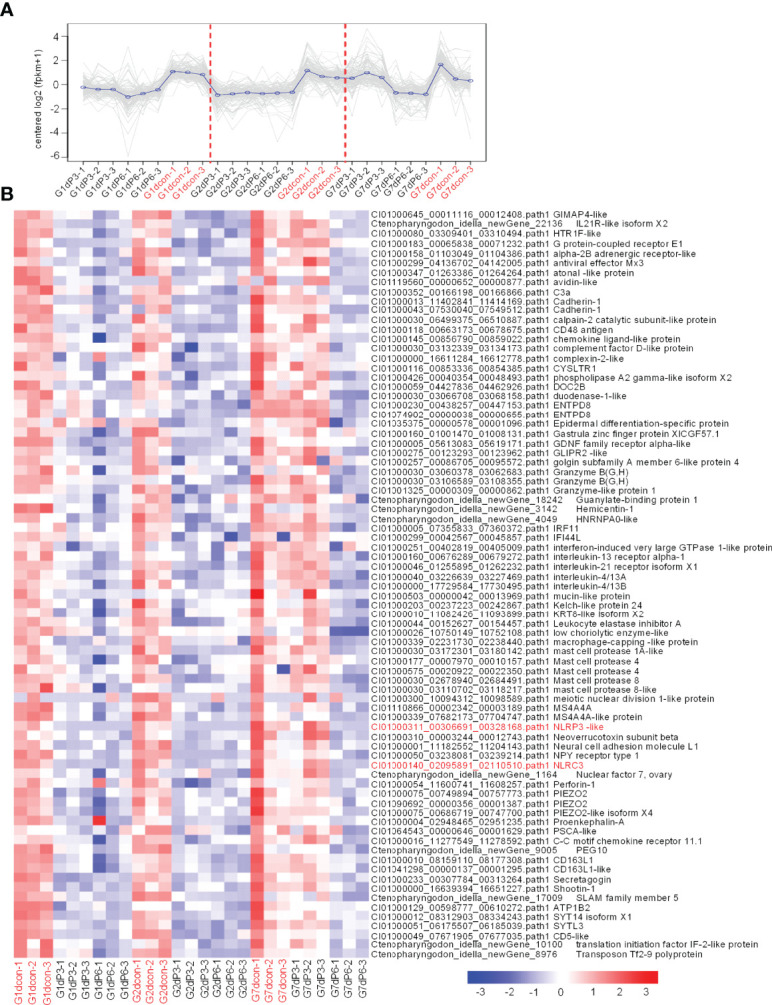
The immune-related DEGs regulated by salinity treatments at all time points of infection. **(A)** The expression trend for DEGs in gills collected at 1, 2 and 7 dpi. The control group at each time point is highlighted in red. **(B)** The immune-related DEGs with the similar expression trend. Those NLR genes are highlighted in red. A color key denotes the gradient scale of gene expression from low (blue) to high (red) degrees.

**Figure 7:**
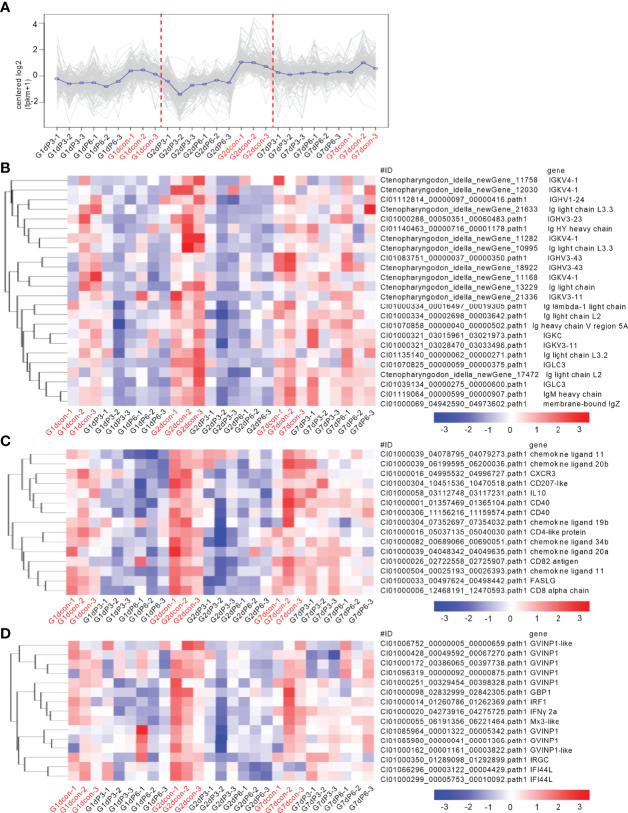
Salinity treatments impair the innate and adaptive immune responses in the case of bacterial infection. **(A)** The expression trend for DEGs in gills collected at 1, 2 and 7 dpi. **(B)** The immune-related DEGs related to immunoglobulin light chain or heavy chain with the similar expression trend. **(C)** The immune-related DEGs related to cytokines or cytokine receptors with the similar expression trend. **(D)** The immune-related DEGs related to interferon responses with the similar expression trend. For **(B–D)**, a color key denotes the gradient scale of gene expression from low (blue) to high (red) degrees. For **(A–D)**, the control group at each time point is highlighted in red.

To reveal the possible mechanisms the grass carp treated by salinity treatments were more vulnerable to the infection of *F. columnare*, analysis of coexpression of differential genes were used for seeking the common immune-related DEGs regulated by salinity treatments at all time points of infection. For the subcluster shown in **Figure 6A**, the transcriptions of 79 DEGs were impaired by salinity treatments, which include 11 cytokines or cytokine receptors, 5 mast cell proteases, 3 Granzyme-like proteins and so on (**Figure 6B**). For the subcluster shown in **Figure 7A**, the transcriptions of 24 DEGs related to immunoglobulin light chain or heavy chain were impaired by salinity treatments (**Figure 7B**), together with 15 cytokines or cytokine receptors (**Figure 7C**) and 15 genes connected to interferon responses (**Figure 7D**).

### Confirmation of DEGs by qRT-PCR and Western Blotting

**Figure 8:**
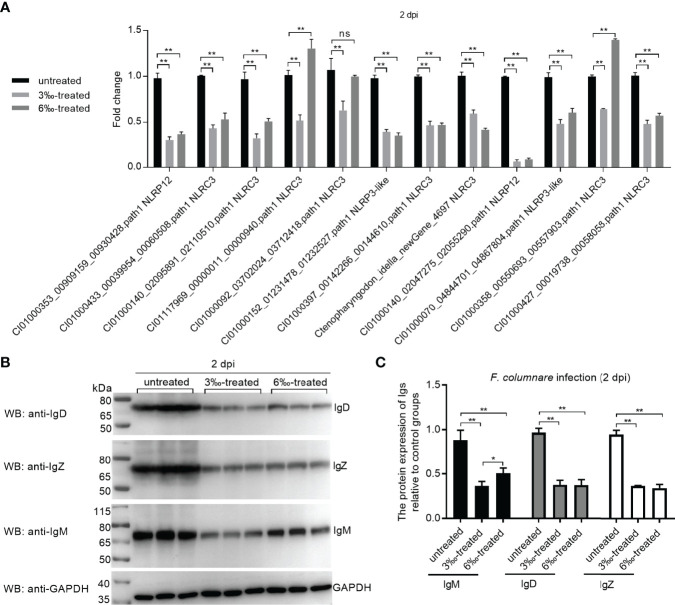
Validation of transcriptome data by qRT-PCR and Western Blotting. **(A)** Validation of transcriptome data by qRT-PCR for differentially expressed NLRs in gills collected at 2 dpi. Data represented means ± SEM (n=3), and were tested for statistical significance. **p < 0.01; ns, not significant. The asterisk above the bracket indicates statistical significance between the two groups connected by the bracket. **(B)** Validation of transcriptome data by Western Blotting for immunoglobulins in gills collected at 2 dpi. **(C)** Gray quantification for Ig protein bands. Western blotting results were quantified using Quantity One software. *p < 0.05; **p < 0.01.

The expression of 12 candidate DEGs was confirmed by qRT-PCR in the untreated control and salinity-treated groups infected with *F. columnare* for 2 d. The expression of 12 NLRs for con at 2 dpi vs 3‰-treatment at 2 dpi agreed with their significant changes determined by RNA-seq. For con at 2 dpi vs 6‰-treatment at 2 dpi, the expressions of CI01000353_00909159_00930428.path1 NLRP12, CI01000140_02095891_02110510.path1 NLRC3, CI01117969_00000011_00000940.path1 NLRC3, CI01000140_02047275_02055290.path1 NLRP12, CI01000070_04844701_04867804.path1 NLRP3-like, CI01000427_00019738_00058058.path1 NLRC3 and CI01000092_03702024_03712418.path1 NLRC3 agreed with their significant changes determined by RNA-seq (**Figure 8A**). In all, 19/24 (79.2%) consistency existed between qRT-PCR and RNAseq. In addition, the protein levels of IgM, IgD and IgZ were examined in gills from the untreated control and salinity-treated groups infected with *F. columnare* for 2 d by Western blotting. Compared with the untreated control, salinity treatments significantly decreased the protein levels of IgM, IgD and IgZ, especially for 3‰ salinity-treated group (**Figures 8B, C**).

### 3‰ Salinity-Treated Group Has a Lower Immune Responses Than 6 ‰ Salinity-Treated Group

**Figure 9:**
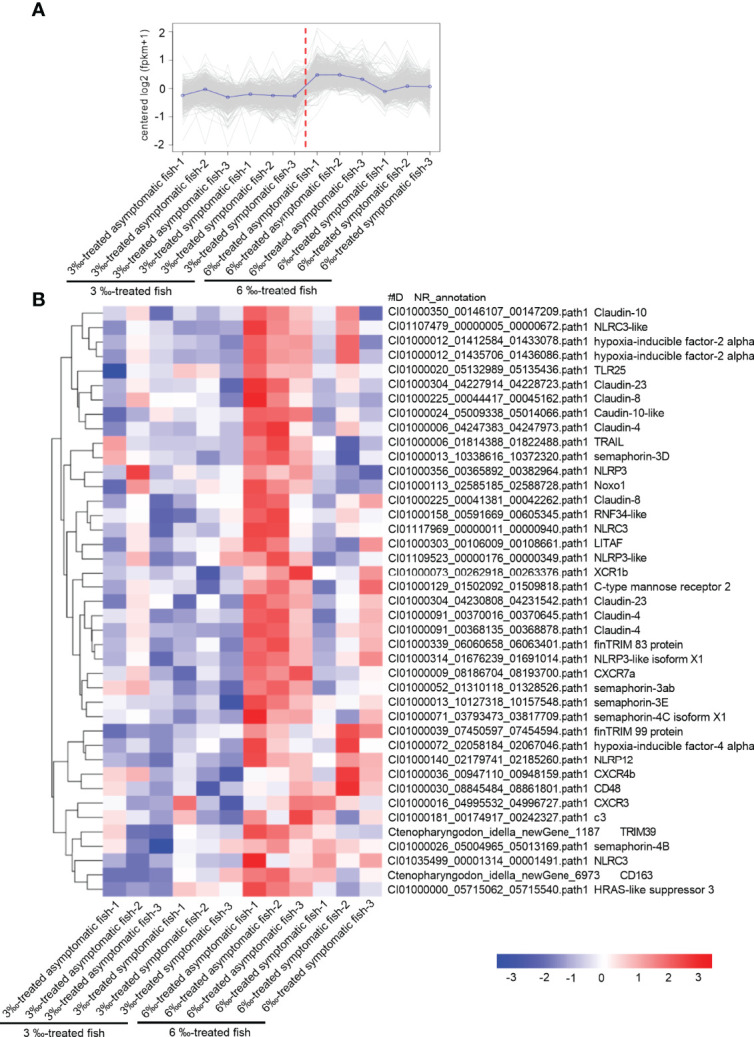
The immune-related DEGs with the lower expressions in 3 ‰ salinity-treated fish than in 6‰ salinity-treated fish. **(A)** The expression trend for DEGs in gills collected from 3‰ and 6‰ salinity-treated fish. **(B)** The immune-related DEGs with the similar expression trend in the 3‰ and 6‰ salinity-treated fish. A color key denotes the gradient scale of gene expression from low (blue) to high (red) degrees.

Compared with 6‰ salinity-treated fish, 3‰ salinity-treated fish have more severe symptoms, the higher percent of unhealthy fish, and higher susceptibility to *F. columnare* infection. To clarify the possible mechanisms why the severity of pathological symptoms and mortality of grass carp treated with 6‰ were much lower than those treated with 3‰, comparative transcriptome analysis were performed for 3‰-treated asymptomatic fish, 3‰-treated symptomatic fish, 6‰-treated asymptomatic fish and 6‰-treated symptomatic fish. For the subcluster shown in **Figure 9A**, the transcriptions of 41 immune-related DEGs were lower in 3‰-treated fish than in 6‰-treated fish, which include 7 NLRs (2 NLRC3, 1 NLRC3-like, 1 NLRP3, 1 NLRP3-like, 1 NLRP3-like isoform X1 and 1 NLRP12), 9 claudin proteins (3 claudin-4, 2 claudin-8, 1 claudin-10, 1 claudin-10-like, 2 claudin-23), 5 semaphorins (semaphoring-3D, semaphoring-3ab, semaphoring-3E, semaphoring-4C isoform X1 and semaphoring-4B), 6 cytokines or cytokine receptors (XCR1b, CXCR7a, CXCR4b, CXCR3, CD48, CD163), 3 hypoxia-inducible factors (2 hypoxia-inducible factor-2 alpha and 1 hypoxia-inducible factor-4 alpha), 3 TRIM proteins (finTRIM 83, finTRIM99 and TRIM39) and so on (**Figure 9B**).

### Two NLRP12-Like Genes Impaired by Salinity Treatments Contribute to Inhibit the Proliferation of *F. columnare*

**Figure 10:**
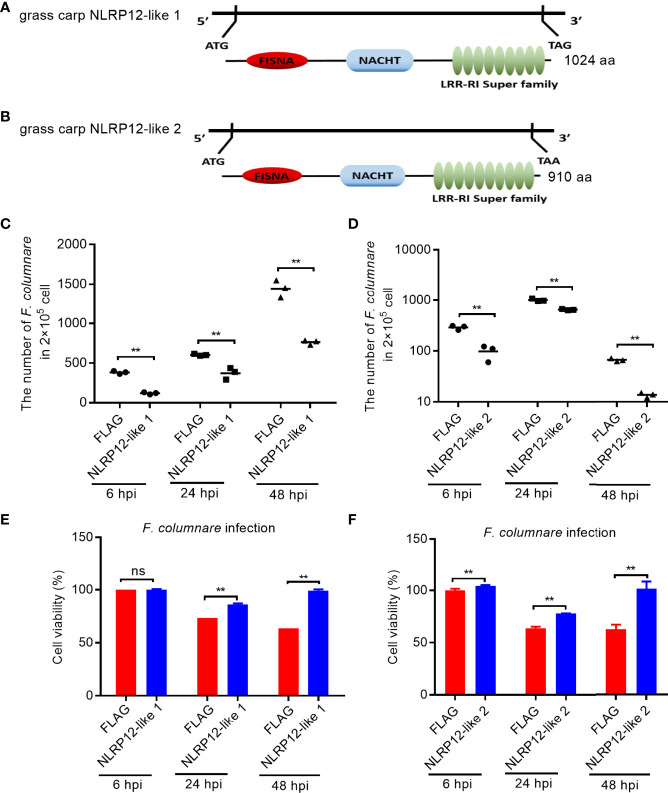
The effects of NLRP12-like genes impaired by salinity treatments in bacterial infection. **(A)** Domain diagram for grass carp NLRP12-like 1. **(B)** Domain diagram for grass carp NLRP12-like 2. **(C)** The effect of grass carp NLRP12-like 1 on the *F*. *columnare* proliferation. **(D)** The effect of grass carp NLRP12-like 2 on the *F*. *columnare* proliferation. **(E)** The effect of grass carp NLRP12-like 1 on the cell survival. **(F)** The effect of grass carp NLRP12-like 2 on the cell survival. For **(C–F)**, Data represented means ± SEM (n = 3), and were tested for statistical significance. **p < 0.01; ns, not significant. The asterisk above the bracket indicates statistical significance between the two groups connected by the bracket.

Salinity treatments significantly enriched NOD-like receptor signaling pathway, and regulated the transcription levels of a large number of NLR receptor genes, especially for piscine NLRC3-like and NLRP-like genes, whose functions are unknown. To screen and identify the target genes of sensing environment salinity stress and pathogens, we were interested to know whether these NLRs regulated by salinity have a function in the regulation of antibacterial or antiviral immune responses. At present, we cloned and obtained 2 NLRP-like genes. Among them, the gene corresponding to Ctenopharyngodon_idella_newGene_9152 was named as NLRP12-like 1 (GenBank accession number: ON012777). The complete ORF of NLRP12-like 1 encodes 1024 aa, which contains 1 N-terminal FISNA, 1 NACHT and 9 LRR domains (**Figure 10A**). The gene corresponding to CI01000353_00909159_00930428.path1 was named as NLRP12-like 2 (GenBank accession number: ON012778). The complete ORF of NLRP12-like 2 encodes 910 aa, which contains 1 N-terminal FISNA, 1 NACHT and 10 LRR domains (**Figure 10B**).

To evaluate the role of NLRP12-like 1 and NLRP12-like 2 on the *F. columnare* proliferation and cell survival, we performed antibacterial assay and CCK8 assay. Compared with control cells transfected with FLAG empty plasmid, the overexpression of NLRP12-like 1 or NLRP12-like 2 in CIK cells significantly inhibited the proliferation of *F. columnare* at 6, 24 and 48 hpi (**Figures 10C, D** **)**, and increased cell survival at 24 and 48 hpi (**Figures 10E, F** **)**.

## Discussion

The development and utilization of fish culture in saline-alkali water is an important way to improve comprehensive production capacity of saline-alkali water. Grass carp is an economically important freshwater fish in china, which can tolerate low levels of salinity and even inhabit brackish water (19, 20). Previous studies have shown the effects of salinity on the electrocardiogram and some of blood serum minerals (20), thyroid activity (21), serum total protein (22), the behavior and physiology of grass carp (23). After 30 days of temporary rearing in brackish water, the salinity of 5‰ and 7.5‰ had no effect on the weight gain rate and survival rate of grass carp (24). In the present study, we report the effects and molecular regulation mechanisms of different concentrations of salinity stress on the health and disease resistance of grass carp. Similar to previous report (24), salinity treatments using 3‰ or 6‰ concentrations had no effect on the survival rate of grass carp in the absence of infection. However evaluation of the percent of unhealthy fish in the absence of infection and the survival of grass carp infected with *F. columnarae* showed that salinity treatments significantly impaired the health and disease resistance of grass carp. Interesting, the severity of pathological symptoms and mortality of grass carp treated with 6‰ were much lower than those treated with 3‰, which was different from this phenomenon observed in euryhaline teleosts. The fish cultured in low salinity present better growth performances and survival rates than in normal salinity in large yellow croaker *Larimichthys crocea*, grey mullet *Mugil cephalus* and pompano *Trachinotus marginatus* (25–27). These differences may reflect the existence of different adaptive mechanisms to salinity stress between the marine and freshwater fish species.

For immune-related signaling pathways, none was annotated in the gills of tiger puffer (*Takifugu rubripes*) with the low-salinity stress of 4‰ (28), cytokine-cytokine receptor interaction (41 DEGs) annotated in the gills of hybrid tilapia with the salinity stress of 25‰ (29), TNF signaling pathway (60 DEGs), NF-kappa B signaling pathway (55 DEGs) and cytokine-cytokine receptor interaction (70 DEGs) annotated in the gills of Siberian sturgeon *Acipenser baeri* with the high-salinity stress of 30‰ (30). In the present study, higher numbers of DEGs involved in cytokine-cytokine receptor interaction were enriched in the gills of grass carp treated with 6‰ salinity (64 DEGs) than in the gills of grass carp treated with 3‰ salinity (36 DEGs) in the absence of infection. All these data suggest that the higher salinity need recruit the greater numbers of cytokines and their receptors to balance inflammatory response, since cytokines play a role in a variety of inflammatory processes (31). Besides that, these DEGs involved in Toll-like receptor signaling pathway and apoptosis were only impaired in the 6‰-treated asymptomatic fish. Most genes involved in Toll-like receptor signaling pathway are cytokines and cytokine receptors such as chemokine ligand 8b, chemokine ligand 11, chemokine ligand 18a, chemokine ligand-like protein, tumor necrosis factor-alpha, interleukin-8 and so on. For these DEGs related to apoptosis, the expressions of calpain-1, calpain-2, calpain-8, cathepsin B, and tumor necrosis factor-alpha so on were decreased. In the pacific oyster *Crassostrea gigas* and the white shrimp *Litopenaeus vannamei*, it is believed that apoptosis suppression may be one of reasons that they are able to deal with large variations in salinity as euryhaline organisms (32, 33). We speculate that apoptosis suppression might contribute to the salinity adaptation of grass carp in the salinity water of 6‰ more than in 3‰. Further research is needed to confirm how apoptosis suppression affect the survival of freshwater fishes in different saline-alkali water.

NOD-like receptor signaling pathway is one of crucial signaling pathways for in host defense against pathogen infection. Recent studies have shown that NOD-like receptor signaling pathway is significantly enriched in silvery pomfret under high salinity stress (32‰), and in all salinity comparison groups including 14‰ vs 22‰, 22‰ vs 30‰ and 14‰ vs 30‰ for the ark shell *Anadara kagoshimensis* (13, 34). In grass carp, NOD-like receptor signaling pathway is significantly enriched in 6‰-treated asymptomatic fish but not in 3 ‰-treated asymptomatic fish, which may suggest that in the absence of infection, NOD-like receptor signaling pathway can only sense salinity stress above a certain level. Salinity treatments can trigger an inflammatory response in a very small amount of grass carp, as evidenced by the increased NLRP1, NLRP3, NLRP12, pro-IL-1β and IL-1β (**Supplementary Figure 1**). Different from salinity-treated symptomatic fish, more DEGs involved in the upstream sensors of NOD-like receptor signaling pathway, which include 32 NLRs (NOD1, CARD, NLRC3-like proteins, NLRP1-like proteins, NLRP12-like proteins), anthrax toxin receptor 2 (ANTXR), RNA helicase DHX33, and GPCR, were induced in the gills of 6‰-treated asymptomatic fish (**Supplementary Figure 2**). In addition, compared with 3‰-treated asymptomatic and symptomatic fish, the expressions of 7 NLRs were significantly higher in 6‰-treated asymptomatic and symptomatic fish. We speculate that these NLRs regulated by salinity treatments may act as the sensors for environmental salinity stress. How these NLRs affect grass carp adaptation to salinity stress remain to study further.

It is generally believed that all NLRs have the common domains of NACHT-LRR. However unlike NLRC subgroup in mammals, these NLRs with the FISNA (Fish-specific NACHT associated) domain but not caspase recruitment domain is considered to be unique for teleost fish (35). Interesting, these NLRs unique for teleost fish, were regulated by salinity and *F. columnare* infection. The function of most NLRs unique for teleost fish is unknown. Our previous study has shown the negative regulation of NLRC3-like 1 in the *Edwardsiella piscicida* infection. Zebrafish NLRC3-like 1 can interact with the adaptor protein RIPK2 of NOD-like receptor signaling pathway *via* its FISNA and NACHT domains, and inhibit the assembly of the NOD1-RIPK2 complex (36). Although the exact functional mechanisms remain to be further investigated, the present study showed that two novel NLRs unique for teleost fish, whose expression levels were significantly impaired by *F. columnare* infection in the grass carp treated with salinity treatments, could inhibit *F. columnare* proliferation and improve the survival rate of infected cells. All these dada revealed that these NLRs unique for teleost fish have different functions in pathogen infection. Furthermore, previous studies showed that transfer from fresh water to seawater did not alter plasma levels of immunoglobulin M (IgM) in the tilapia (*Oreochromis mossambicus*), however salinity ≥20‰ downregulated the mRNA expression levels of IgM and reduced antibody production in the vaccinated Nile tilapia (*O. niloticus*) (37, 38). Compared with the full seawater (38‰)-acclimated fish, the total IgM levels of seabream was increased for the acclimation to hypersaline water (55‰) for 14 days, but no changes for brackish water acclimation (12‰) for 14 or 100 days (39). The results of the present study were consistent with a previous study, which showed a suppressed immune response in freshwater fish in response to salinity treatment and bacterial infection (38). In the absence of infection, salinity treatments have no effects on grass carp IgM, IgD, IgZ, Ig light chain or heavy chain. In the existence of pathogen, salinity treatments significantly impaired the production of Ig levels, especially IgD and IgZ. Significantly, the IgM levels in grass carp left untreated or treated with 3 ‰ and 6 ‰ salinity were consistent with the survival of grass carp with the infection of *F. columnare* (untreated control group > 6‰-treated group > 3‰-treated group). Thus, optimum salinity range may be an important factor in activating the innate and adaptive immune pathways to achieve maximum antibody production, and the inhibited IgM and NLRs may correlate with mortality in grass carp following *F. columnare* infection.

The possible mechanisms why the severity of pathological symptoms and the percent of unhealthy grass carp treated with 6‰ were much lower than those treated with 3‰ were also further explored. Besides ionic and osmotic stress, salinity stress can induce secondary stresses, especially oxidative stress in teleost (40). However oxidative stress plays an essential role in the pathogenesis and progression of inflammatory disease such as inflammatory bowel disease (41). The tight junction (TJ) claudin proteins and hypoxia-inducible factors are involved in the regulation of oxidative stress and intestinal inflammation (42, 43). The downregulations of several “tightening” TJ proteins like claudin-1 and -4, together with an upregulation of claudin-2, were found to contribute to the barrier defect observed in ulcerative colitis (44). Hypoxia-inducible factor-2 alpha was found to play a protective role against ischemia of the kidney *via* amelioration of oxidative stress (45). A conditional knockout of myeloid hypoxia-inducible factor-1 alpha ameliorated whereas the knockout of hypoxia-inducible factor-2 alpha aggravated murine dextran sodium sulfate-induced colitis (46). Here, comparative transcriptome analysis revealed that 3‰ salinity-treated fish has a lower transcriptional levels for 9 claudin proteins, 2 hypoxia-inducible factor-2 alpha and 1 hypoxia-inducible factor-4 alpha. In addition, semaphorins have been identified as the “immune semaphorins” (47). Among them, semaphorin 3A is important in downregulating autoimmune diseases, semaphorin 3E as a critical factor for protective immunity against intracellular *chlamydia muridarum* infection, and semaphorin 4C in protecting against allergic inflammation (48–50). The expressions of these “immune semaphorins” in 6‰ salinity-treated fish were higher than in 3 ‰ salinity-treated fish. Therefore, the difference of immune response involved in oxidative stress and inflammation might lead to the difference in the severity of pathological symptoms and the percent of unhealthy grass carp between 3‰ and 6‰ salinity-treated grass carp.

In short, this study characterized the pathological changes and transcriptomic responses in the gills of grass carp treated with 3‰ and 6‰ salinity. Through the expression data from 5 libraries in the absence of infection and 9 libraries with the infection of *F. columnare*, the present study provided some useful insights into the immune responses of grass carp in response to 3‰ and 6‰ salinity stress, and offered a number of candidate genes as potential markers of sensing environmental salinity stress and pathogens, especially for NLRs. In future work, we will further investigate the exact mechanisms how NLRs unique for teleost fish affect grass carp adaptation to salinity stress and disease resistance against pathogens.

## Data Availability Statement

The datasets presented in this study can be found in online repositories. The names of the repository/repositories and accession number(s) can be found below: NCBI, accession ID: GSE185170 and GSE185641.

## Ethics Statement

All animal experiments were conducted in accordance with the Guiding Principles for the Care and Use of Laboratory Animals approved by the Institute of Hydrobiology, Chinese Academy of Sciences.

## Author Contributions

MC conceived and designed the experiments. HF, XW, YY, SZ, and JZ performed the experiments and analyzed the data. MC and JZ wrote the manuscript. MC revised the manuscript. All authors contributed to the article and approved the submitted version.

## Funding

This study was financially supported by the National Key Research and Development Program of China (Grant No. 2019YFD0900703).

## Conflict of Interest

The authors declare that the research was conducted in the absence of any commercial or financial relationships that could be construed as a potential conflict of interest.

## Publisher’s Note

All claims expressed in this article are solely those of the authors and do not necessarily represent those of their affiliated organizations, or those of the publisher, the editors and the reviewers. Any product that may be evaluated in this article, or claim that may be made by its manufacturer, is not guaranteed or endorsed by the publisher.
