# Supplementary figures and images for: Effects and Molecular Regulation Mechanisms of Salinity Stress on the Health and Disease Resistance of Grass Carp
Source: Front Immunol. 2022 Jun 6;13:917497. doi: 10.3389/fimmu.2022.917497 (PMC9207326; doi:10.3389/fimmu.2022.917497)

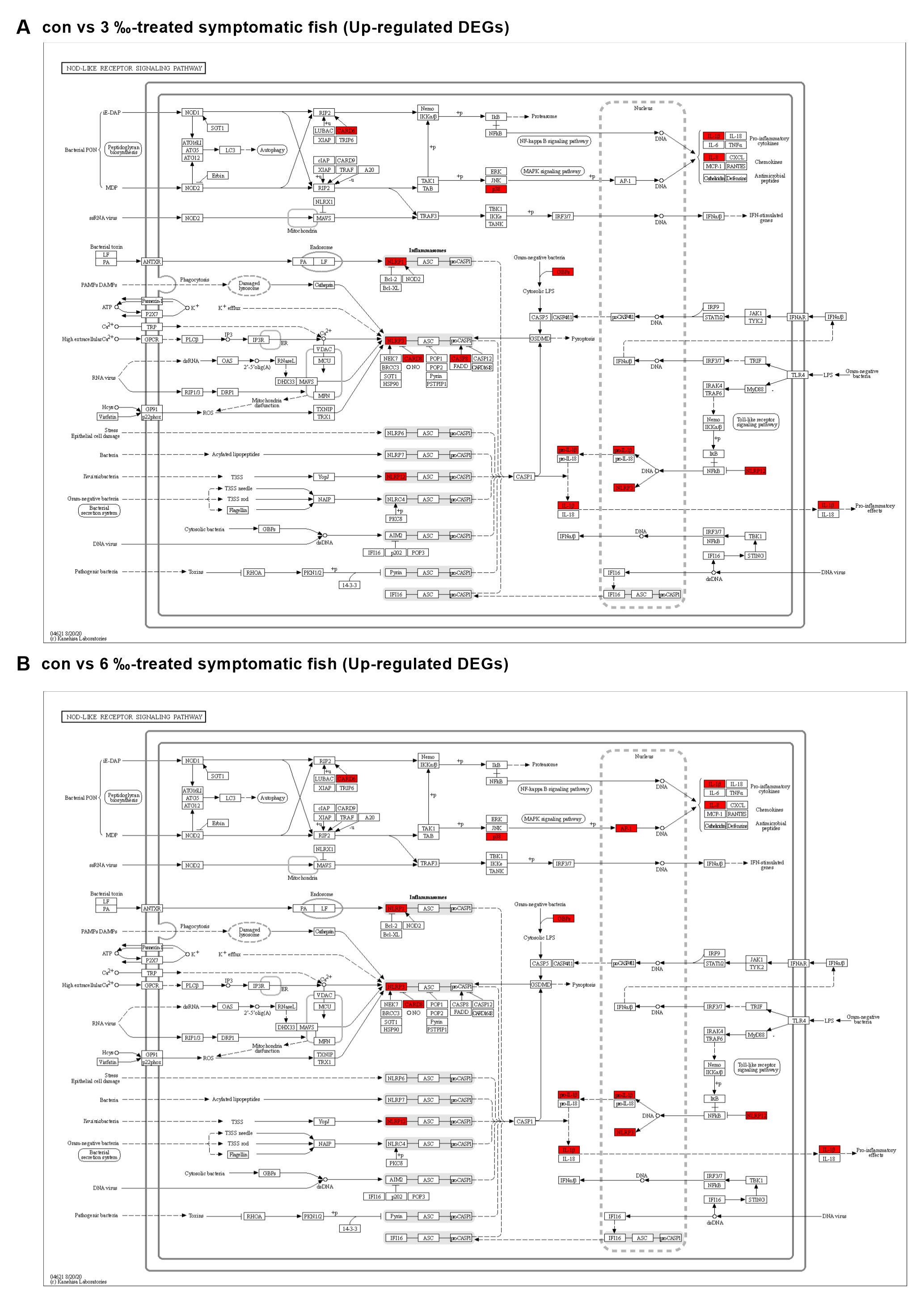

Supplement: Supplementary file 1 [file Image_1.tif]

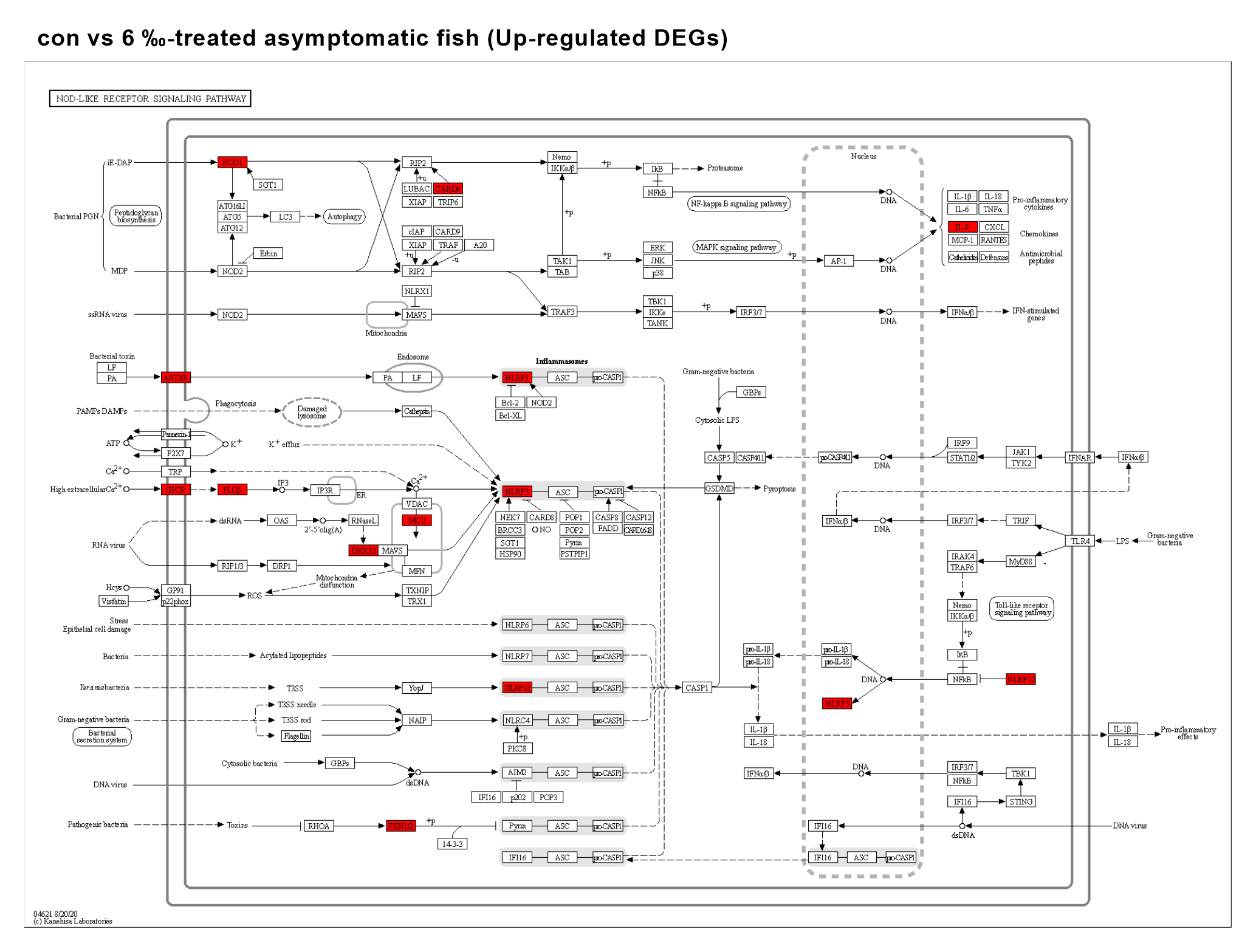

Supplement: Supplementary file 2 [file Image_2.tif]
